# Hepatitis B virus infection: An insight into the clinical connection and molecular interaction between hepatitis B virus and host extrahepatic cancer risk

**DOI:** 10.3389/fimmu.2023.1141956

**Published:** 2023-03-01

**Authors:** Yu Min, Xiaoyuan Wei, Xi Xia, Zhigong Wei, Ruidan Li, Jing Jin, Zheran Liu, Xiaolin Hu, Xingchen Peng

**Affiliations:** ^1^ Department of Biotherapy and National Clinical Research Center for Geriatrics, Cancer Center, West China Hospital, Sichuan University, Sichuan, China; ^2^ Department of Head and Neck Oncology, Department of Radiation Oncology, Cancer Center, and State Key Laboratory of Biotherapy, West China Hospital, Sichuan University, Sichuan, China; ^3^ Research and Development Department Shanghai ETERN Biopharma Co., Ltd., Shanghai, China; ^4^ West China School of Nursing, West China Hospital, Sichuan University, Sichuan, China

**Keywords:** hepatitis B virus, extrahepatic cancer, immune microenvironment, risk factor, molecular mechanisms

## Abstract

The evidence for chronic hepatitis B virus (HBV) infection and hepatocellular carcinoma (HCC) occurrence is well established. The hepatocyte epithelium carcinogenesis caused by HBV has been investigated and reviewed in depth. Nevertheless, recent findings from preclinical and observational studies suggested that chronic HBV infection is equally important in extrahepatic cancer occurrence and survival, specifically gastrointestinal system-derived cancers. Immune microenvironment changes (immune-suppressive cytokine infiltration), epigenetic modification (N6-methyladenosine), molecular signaling pathways (PI3K–Akt and Wnt), and serum biomarkers such as hepatitis B virus X (HBx) protein are potential underlying mechanisms in chronic HBV infection-induced extrahepatic cancers. This narrative review aimed to comprehensively summarize the most recent advances in evaluating the association between chronic HBV infection and extrahepatic cancer risk and explore the potential underlying molecular mechanisms in the carcinogenesis induction of extrahepatic cancers in chronic HBV conditions.

## 1 Introduction

Hepatitis B (HBV) infection is one of the leading global public health issues to date. There are approximately 316 million hepatitis B surface antigen (HBsAg)-seropositive carriers, specifically in the developing and poor countries (1, 2). The serum indicators, which include HBsAg, hepatitis B core antigen (HBcAg), hepatitis B e antigen (HBeAg), hepatitis B virus X (HBx) protein, and HBV DNA copies, are crucial in HBV-associated diseases. The correlation between chronic HBV and hepatocellular carcinoma (HCC) has been investigated in depth. The “three steps” of chronic hepatitis, liver cirrhosis, and HCC have been validated (2–4).

**Figure 1:**
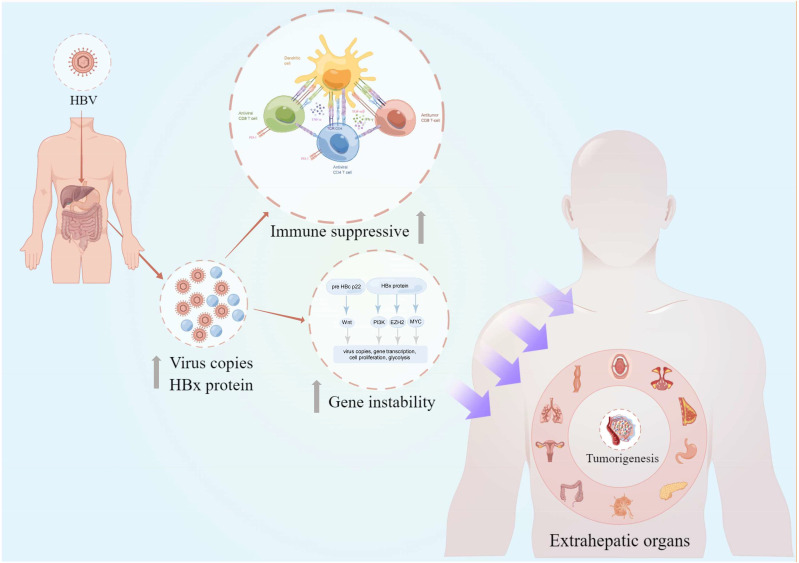
The overview of chronic HBV-mediated extrahepatic cancers in this review. The intrahepatic and extrahepatic HBV copies and HBx protein secretion could lead to the gene instability and host immunosuppressive condition. These adversely effects would further increase the risk of extrahepatic cancer risks in different organs including the oral cavity, nasopharynx, breast, stomach, pancreas, lymph, colorectum, cervix, lung, and esophagus (clockwise description). HBV, hepatitis B virus; pre HBc p22, pre-hepatitis B core protein 22; HBx, hepatitis B virus X; TGF-β, transforming growth factor-β; TNF-α, tumor necrosis factor-α; IFN-γ, interferon γ; PD-1, programmed cell death protein 1; This figure was drawn with the tool “Figdraw” by Dr. Yu Min and Xiaoyuan Wei. HBV, hepatitis B virus.

Emerging evidence also identified abnormal HBV DNA copies in several extrahepatic tissues, including the stomach, pancreas, and colorectum, which indicated the potential association between HBV infection and extrahepatic cancer risk (5, 6). An epidemiological report evaluating the cancer deaths and cases in China attributable to lifestyle factors and infections revealed that HBV infection accounted for 12% and 7% of cancer-associated mortality in men and women, respectively (7). Recent large-scale population-based studies and meta-analyses have detailed these under-estimated connections (8–12). For example, a Chinese prospective cohort study reported that the HBsAg-seropositive population had a significantly increased stomach cancer, colorectal cancer, oral cancer, pancreatic cancer, and lymphoma risk as compared with the HBsAg-seronegative population (13). Furthermore, US health care organizations reported HBV-induced extrahepatic cancers in the US population in 2006–2018 (14). Therefore, assessments of the clinical connections and underlying mechanisms in HBV infection-induced extrahepatic cancers are equally pivotal as they could aid understanding of the HBV biological characteristics in human tissue carcinogenesis and aid individualized monitoring and treatment modalities in the HBV-infected population. In this review, we summarize the most recent progress in chronic HBV infection and extrahepatic cancers and review the possible underlying mechanisms of these connections (**Figure 1**).

## 2 Epidemiology of chronic hepatitis B

Currently, HBV infection is a major global public health concern (15, 16). Approximately 30% of the global population exhibits serological evidence of current or previous HBV infection (1, 17). Vertical transmission (from mother to neonate) and horizontal transmission (mother to child and child to child) remain the main HBV transmission routes, while the administration of contaminated blood products remains a major transmission mode, particularly in the low- and middle-income countries (18, 19).

**Figure 2:**
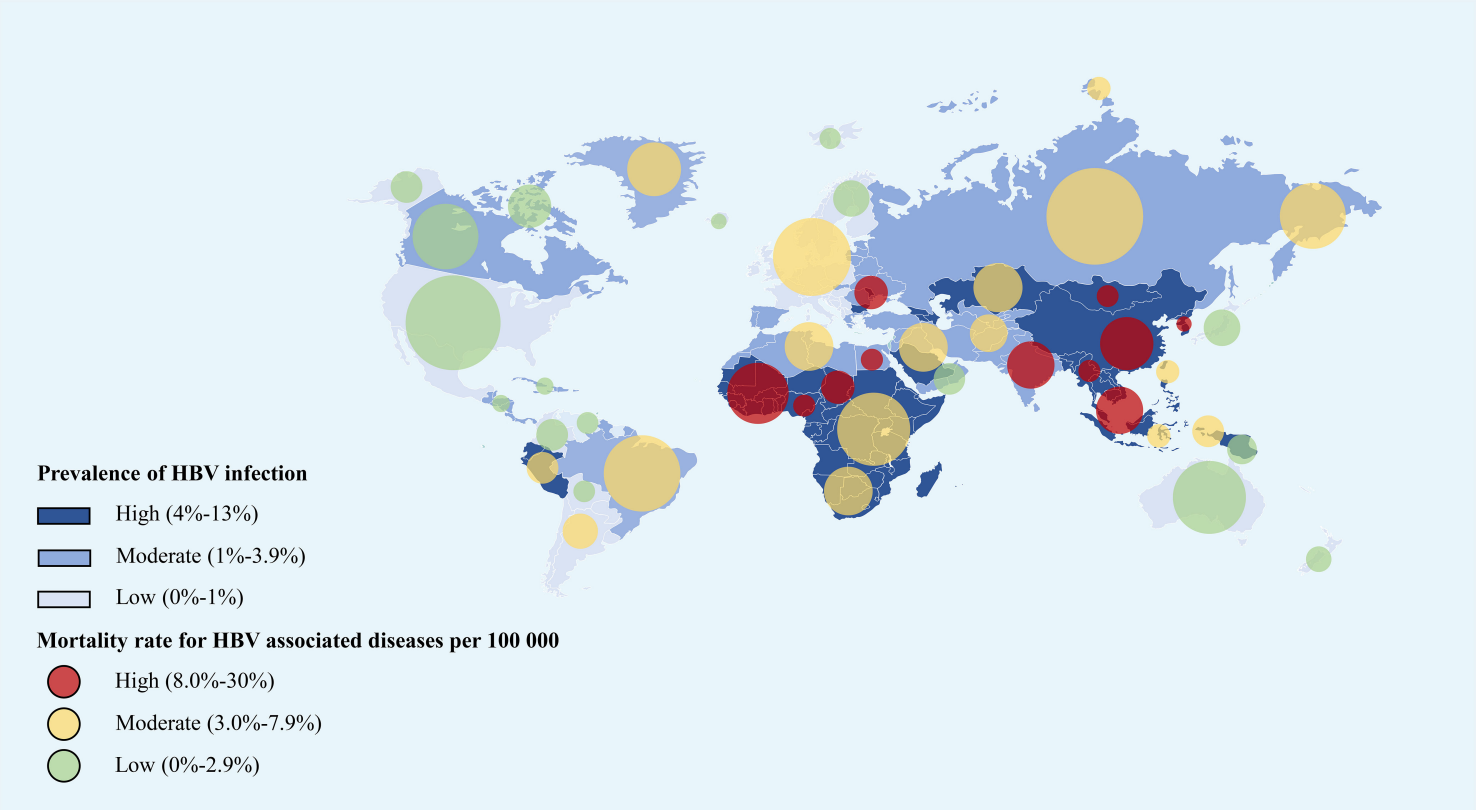
The global prevalence of HBV infection and all-age death rate for HBV associated diseases per 100,000 in 2019 (refers to the Global Burden of Disease Study 2019) (1). The east Asian countries and African countries showed the highest HBV infection rate as well as the mortality rate around the world. This figure was drawn with the tool of “Smart Art” by Dr. Xiaoyuan Wei. HBV, hepatitis B virus.

Since the 1990s, The World Health Organization (WHO) has called for the formulation of several global public plans (routine HBV vaccine injection, fast virus testing, advanced antiviral therapies) to prevent HBV infection and HBV-related disease progression (1, 20–25). Most recently, the global HBV infection burden was assessed by Global Burden of Diseases, Injuries, and Risk Factors Study (GBD) 2019 collaborators (1) (**Figure 2**). In detail, the estimated global all-age prevalence of chronic HBV infection was 4.1% (95% uncertainty interval [UI]: 3.7–4.5), which corresponded to approximately 316 million infected people (95%UI: 284–351). With the wide utility of HBV vaccination in different regions, all-age HBV infection prevalence of the whole population has declined by 31.3% (95%UI: 29.0–33.9%) during the past three decades. Furthermore, a more significant decline in HBV infection prevalence was observed in younger children (<5 years) (76.8%, 95%UI: 76.2–77.5%) during the same period (1). However, HBV infection and associated diseases burden and progress across the world were markedly disparate, where the developing and poor countries predominated (1, 16, 21, 23, 26, 27). HBV infection has been endemic in China since the 1980s, where the infant vaccination rate was 99.5% and the timely birth dose vaccination rate was 96% as compared with that in 1992 (28). Subsequently, the national hepatitis B immunization program led to chronic HBV infections decreasing by 33.9% (28). While significantly decreased HBV infection prevalence was reported in most countries and regions owing to HBV vaccination in the general population, the number of remaining HBV-infected patients continues to be substantial and they require early detection, more precise management, and reduction of further transmission.

## 3 Chronic hepatitis B in extrahepatic cancer risk

The correlation between chronic HBV infection and HCC development has been well investigated (29–32). The integration of virus DNA and the oncoprotein HBx, intrahepatic immune imbalance, and HBV infection-induced cirrhosis are acknowledged as the frequent pivotal factors of hepatocarcinogenesis (3, 33, 34). More recently, epidemiological evidence demonstrated a potential association between HBV infection and extrahepatic cancer risk, which includes gastrointestinal cancers, head and neck cancer, cervical cancer, non-Hodgkin lymphoma, breast cancer, and non-small cell lung cancer (NSCLC) (13, 14, 35–39) (**Figure 1**). Notably, HBV infection status can not only increase the extrahepatic cancer risk but can also significantly influence subsequent long-term survival patterns (40–45). However, a multi-center study demonstrated that a large proportion of patients with newly diagnosed cancer and concurrent HBV were unaware of their viral infection at the time of cancer diagnosis (46). Therefore, exploring the association between chronic HBV infection and pan-cancer could provide more robust evidence to establish effective public health strategies to guide HBV-infected population surveillance. **Table 1** summarizes the recent clinical studies that evaluated the association between chronic HBV infection and extrahepatic cancers.

**Table 1:** The evaluation of associations between chronic HBV infection and extrahepatic cancers in recent studies.

Cancer type	First author	Year	Country	Sample size (No.)	Study design	Main results	Reference
Gastric cancer							
	Mahale et al.	2019	USA	34,412 cases of gastric cancer and 200,000 cancer-free controls	Case-control	OR= 1.19 (1.03-1.37)	(47)
	Wei et al.	2017	China	2,318 cases of gastric cancer and 5,715 non-cancer controls	Case-control	OR= 1.24 (1.06-1.45)	(48)
	Wei et al.	2015	China	580 cases of gastric cancer and 580 controls	Case-control	OR= 1.49 (1.06-2.10)	(49)
	An et al.	2018	Korea	16,350 cases of gastric cancer and 118,891 healthy controls	Case-control	OR= 1.03 (0.90–1.17) in men OR= 0.98 (0.79–1.21) in women	(8)
	Song et al.	2019	China	15,355 cases with HBsAg seropositive and 481,377 cases with HBsAg seronegative in CKB	Cohort	HR= 1.41 (1.11-1.80)	(13)
	Song et al.	2019	China	3,539 cases with HBsAg seropositive and 33,797 cases with HBsAg seronegative in Qidong Cohort	Cohort	HR= 2.02 (1.24-3.29)	(13)
	Baghbanian et al.	2019	Iran	35 cases of gastric cancer and 259 normal controls	Case-control	OR= 2.127 (0.242-18.704)	(50)
	Wang et al.	2020	China	7 studies with 378,854 cases	Meta-analysis	OR= 1.39 (1.11-1.75)	(51)
	Cui et al.	2019	China	268 cases of gastric cancer and 420 healthy controls	Case-control	HBsAg seropositive rate: 10/258 *vs*. 20/400	(52)
	Yang et al.	2021	China	10 studies with 7,027,546 cases	Meta-analysis	HR= 1.26 (1.08-1.47)	(53)
	Liu et al.	2022	China	2,607 cases with HBsAg seropositive and 90,795 cases with HBsAg seronegative	Cohort	HR= 1.20 (0.64-2.25)	(35)
	Lu et al.	2018	China	961 cases with HBsAg seropositive and 5,986 cases with HBsAg seronegative	Cohort	OR= 1.763 (1.424–2.169) in 0–39 years OR= 1.284 (1.120–1.472) in 40–59 years OR= 0.631 (0.549–0.725) in ≥60 years	(12)
	Spradling et al.	2022	USA	5,773 HBV cases and general healthy control	Cohort	SRR= 7.95 (7.71–8.20)	(14)
Pancreatic cancer							
	Liu et al.	2022	China	2,607 cases with HBsAg seropositive and 90,795 cases with HBsAg seronegative	Cohort	HR= 1.86 (1.10-3.99)	(35)
	Huang	2013	Sweden	5 cases of HBV infection and 16 control	Cohort	HR= 1.80 (0.70-5.10)	(54)
	Tang	2014	USA	404 cases with HBV infection and 28,719 control	Cohort	HR= 1.08 (0.15-7.76)	(55)
	Chang	2014	Taiwan	585 cases and 1716 controls	Case-control	OR= 0.89 (0.89-1.68)	(56)
	Desai et al.	2018	USA	175 patients with a history of HBV and 69,035 patients without a history of HBV	Cohort	HR= 1.24 (1.056-1.449)	(57)
	Fiorino et al.	2013	Italy	5 studies with 29,142 cases	Meta-analysis	RR= 1.18 (1.04-1.33)	(58)
	Xu et al.	2013	China	4 studies with 657118 cases	Meta-analysis	OR= 1.20 (1.01-1.39)	(59)
	Batskikh et al.	2022	Russian	60 cases and 70 control	Case-control	OR= 2.905 (1.191-7.084)	(36)
	Xing et al.	2013	China	9 studies with 157,275 cases	Meta-analysis	OR= 1.28 (1.11-1.48)	(60)
	Spradling et al.	2022	USA	5,773 HBV cases and general healthy control	Cohort	SRR= 1.36 (1.34–1.38)	(14)
	An et al.	2018	Korea	3,622 cases of pancreatic cancer and 118,891 healthy controls	Case-control	OR= 1.17 (0.84-1.62) in men OR= 0.93 (0.60–1.44) in women	(8)
	Song et al.	2019	China	15,355 cases with HBsAg seropositive and 481,377 cases with HBsAg seronegative in CKB	Cohort	HR= 1.65 (1.03-2.65)	(13)
	Wei et al.	2017	China	276 cases of pancreatic cancer and 5,715 non-cancer controls	Case-control	OR= 1.42 (1.00-2.01)	(48)
Colorectal cancer							
	Liu et al.	2022	China	2,607 cases with HBsAg seropositive and 90,795 cases with HBsAg seronegative	Cohort	HR= 1.75 (1.15-2.96)	(35)
	Spradling et al.	2022	USA	5,773 HBV cases and general healthy control	Cohort	SRR= 1.50 (1.49–1.51)	(14)
	Liu et al.	2021	China	2,598 cases with HBsAg seropositive and 90,792 cases with HBsAg seronegative	Cohort	HR= 1.85 (1.15-2.96)	(61)
	An et al.	2018	Korea	7,022 cases of colorectal cancer and 118,891 healthy controls	Case-control	OR= 1.13 (0.90–1.42) in men OR= 1.15 (0.87–1.52) in women	(8)
	Wei et al.	2017	China	1,828 cases of colorectal cancer and 5,715 non-cancer controls	Case-control	OR= 1.04 (0.87–1.24)	(48)
	Song et al.	2019	China	15,355 cases with HBsAg seropositive and 481,377 cases with HBsAg seronegative in CKB	Cohort	HR= 1.42 (1.12-1.81)	(13)
HNC							
	Lu et al.	2018	China	535 cases with HBsAg seropositive and 2,788 cases with HBsAg seronegative	Cohort	OR= 1.525 (1.238–1.872) in 0–39 years OR= 1.008 (0.836–1.217) in 40–59 years OR= 0.536 (0.402–0.703) in ≥60 years	(12)
	Ye et al.	2015	China	711 NPC cases and 656 individuals with other benign tumors unrelated to HBV infection and 680 healthy population controls	Case-control	OR= 1.40 (1.12 to 1.74) compared with the benign tumors group OR= 1.48 (1.05-2.08) compared with the healthy group	(62)
	An et al.	2018	Korea	1,750 cases with HNC and 118,891 healthy controls	Case-control	OR= 1.33 (0.94–1.88) in men OR= 1.33 (0.63–2.82) in women	(8)
	Wei et al.	2017	China	4,152 cases with HNC and 5,715 non-cancer controls	Case-control	OR= 1.37 (1.21–1.50)	(48)
	Donà et al.	2019	Italy	774 HNSCC cases and 1518 health control	Case-control	OR= 2.76 (1.64-4.64)	(63)
	Komori et al.	2020	Japan	512 HNC cases and 495 health control	Case-control	OR= 1.50 (1.09-2.08)	(64)
	Song et al.	2019	China	15,355 cases with HBsAg seropositive and 481,377 cases with HBsAg seronegative in CKB	Cohort	HR=1.58 (1.01-2.49)	(13)
Lymphoma							
	Wei et al.	2017	China	1,749 cases with NHL and 5,715 non-cancer controls	Case-control	OR= 1.86 (1.61–2.15)	(48)
	An et al.	2018	Korea	2,277 cases with NHL and 118,891 healthy controls	Case-control	OR= 1.53 (1.12–2.09) in men OR= 3.04 (1.92–4.82) in women	(8)
	Lu et al.	2018	China	882 cases with HBsAg seropositive and 3,371 cases with HBsAg seronegative	Cohort	OR= 1.138 (0.972–1.331) in 0–39 years OR= 1.372 (1.179–1.595) in 40–59 years OR= 0.599 (0.503–0.710) in ≥60 years	(12)
	Spradling et al.	2022	USA	5773 HBV cases and general healthy control	Cohort	SRR= 2.52 (2.49–2.55)	(14)
	Li et al.	2020	China	411 NHL patients and 957 healthy controls	Case-control	OR= 3.36 (2.33-4.84) in B-cell NHL OR= 2.13 (0.92-4.92) in T-cell NHL	(37)
	Li et al.	2018	China	58 studies with a total of 53,714 NHL cases and 1 778 591 controls	Meta-analysis	OR= 2.50 (2.20-2.83) in the whole NHL OR= 2.46 (1.97-3.07) in B-cell NHL OR= 1.67 (1.34-2.10) in T-cell NHL	(38)
	Su et al.	2018	Taiwan	203,031 chronic HBV patients and 203,031 patients without HBV	Cohort	HR= 2.18 (1.80‐2.65)	(65)
	Song et al.	2019	China	15,355 cases with HBsAg seropositive and 481,377 cases with HBsAg seronegative in CKB	Cohort	HR= 2.10 (1.34-3.31)	(13)
Esophageal cancer							
	Wei et al.	2017	China	2,253 cases of esophageal cancer and 5,715 non-cancer controls	Case-control	OR= 1.16 (0.97–1.40)	(48)
	Lu et al.	2018	China	501 cases with HBsAg seropositive 3,816 cases with HBsAg seronegative	Cohort	OR= 2.461 (1.033–5.254) in 0–39 years OR= 1.396 (1.159–1.683) in 40–59 years OR= 0.690 (0.572–0.831) in ≥60 years	(12)
	Liu et al.	2022	China	2,607 cases with HBsAg seropositive and 90,795 cases with HBsAg seronegative	Cohort	HR= 1.07 (0.34-3.37)	(35)
	An et al.	2018	Korea	1,151 cases with NHL and 118,891 healthy controls	Case-control	OR= 1.00 (0.62–1.62) in men OR= 0.50 (0.05–5.51) in women	(8)
	Song et al.	2019	China	15,355 cases with HBsAg seropositive and 481,377 cases with HBsAg seronegative in CKB	Cohort	HR= 0.87 (0.62-1.23)	(13)
	Geng et al.	2022	China	10 studies with 142,437 cases and 1,382,254 controls	Meta-analysis	OR= 1.19 (1.01-1.36)	(10)
Breast cancer							
	Wei et al.	2017	China	2,858 cases of breast cancer and 5,715 non-cancer controls	Case-control	OR= 1.02 (0.88–1.20)	(48)
	Lu et al.	2018	China	902 cases with HBsAg seropositive 6,933 cases with HBsAg seronegative	Cohort	OR= 1.487 (1.255–1.755) in 0–39 years OR= 1.049 (0.909–1.213) in 40–59 years OR= 0.592 (0.483–0.720) in ≥60 years	(12)
	Spradling et al.	2022	USA	5,773 HBV cases and general healthy control	Cohort	SRR= 1.46 (1.45–1.47)	(14)
	An et al.	2018	Korea	12,487 cases of breast cancer and 118,891 healthy controls	Case-control	OR= 1.16 (1.02–1.32) in women	(8)
	Song et al.	2019	China	15,355 cases with HBsAg seropositive and 481,377 cases with HBsAg seronegative in CKB	Cohort	HR= 0.86 (0.71-1.29)	(13)
	Song et al.	2019	China	3,539 cases with HBsAg seropositive and 33,797 cases with HBsAg seronegative in Qidong Cohort	Cohort	HR= 1.71 (0.87-3.33)	(13)
Lung cancer							
	Wei et al.	2017	China	5,008 cases of lung cancer and 5,715 non-cancer controls	Case-control	OR= 1.06 (0.93–1.21)	(48)
	An et al.	2018	Korea	8,409 cases of lung cancer and 118,891 healthy controls	Case-control	OR= 0.91 (0.75–1.11) in men OR= 1.79 (1.32–2.44) in women	(8)
	Lu et al.	2018	China	2,335 cases with HBsAg seropositive and 17,277 cases with HBsAg seronegative	Cohort	OR= 1.616 (1.343–1.931) in 0–39 years OR= 1.468 (1.346–1.601) in 40–59 years OR=0.620 (0.568–0.677) in ≥60 years	(12)
	Spradling et al.	2022	USA	5773 HBV cases and general healthy control	Cohort	SRR= 1.17 (1.16–1.18)	(14)
	Song et al.	2019	China	15,355 cases with HBsAg seropositive and 481,377 cases with HBsAg seronegative in CKB	Cohort	HR= 1.07 (0.87-1.31)	(13)
	Song et al.	2019	China	3,539 cases with HBsAg seropositive and 33,797 cases with HBsAg seronegative in Qidong Cohort	Cohort	HR= 1.31 (0.84-2.06)	(13)
Cervical cancer							
	Wei et al.	2017	China	2,470 cases of cervical cancer and 5,715 non-cancer controls	Case-control	OR= 1.22 (1.05–1.42)	(48)
	An et al.	2018	Korea	2,370 cases of cervical cancer and 118,891 healthy controls	Case-control	OR= 1.49 (1.11–2.00) in women	(8)
	Song et al.	2019	China	15,355 cases with HBsAg seropositive and 481,377 cases with HBsAg seronegative in CKB	Cohort	HR= 0.79 (0.47-1.35)	(13)
	Lu et al.	2018	China	2,335 cases with HBsAg seropositive and 17,277 cases with HBsAg seronegative	Cohort	OR= 1.175 (0.926–1.480) in 0–39 years OR= 1.052 (0.861–1.289) in 40–59 years OR= 0.728 (0.538–0.967) in ≥60 years	(12)

HBV, hepatitis B virus; OR, Odd ratio; HR, Hazard ratio; SRR, Standardized rate ratio; HBsAg, hepatitis B surface antigen; CKB, The China Kadoorie Biobank; HNC, head and neck cancer; NPC, Nasopharyngeal carcinoma; HNSCC, head and neck squamous cell carcinoma; NHL, Non-Hodgkin’s lymphoma.

### 3.1 Gastric cancer

Gastric cancer is one of the specific infection‐associated cancers. *Helicobacter pylori* (Hp) and Epstein‐Barr virus (EBV) infections are well-known gastric cancer risk factors (66–68). Recent evidence demonstrated a positive association between chronic HBV infections and gastric cancer risk. A Chinese retrospective study was the first to demonstrate this potential correlation (49). The study involved 580 cases and 580 matched controls, and the authors (49) determined that HBsAg seropositivity was significantly associated with increased gastric cancer risk (adjusted odds ratio [_adjusted_OR] = 1.49, 95% confidence interval [CI]: 1.06–2.10) in the whole group and in patients without a family history of gastric cancer (_adjusted_OR = 1.49, 95%CI: 1.06–2.11). In a pilot study, the relatively limited evidence of the study design and the lack of records on Hp infection could not resolve the question of whether HBV infection patients required different screening plans or additional surveillance for gastric cancer (39). In the prospective Kailuan Cohort Study (HBsAg seronegative: 93,402 cases; HBsAg seropositive: 2,607 cases), Liu et al. (35) comprehensively evaluated the correlation between HBV infection and newly diagnosed gastrointestinal cancers. However, the subgroup analysis results did not support the hypothesis (adjusted hazard ratio [_adjusted_HR] = 1.20, 95%CI: 0.64–2.25). Conversely, the results from another Chinese hospital experience (52) revealed no association between HBV infection and gastric cancer risk. Nonetheless, histological examination revealed an increased nuclei‐to‐cytoplasm ratio in the HBV-exposed gastric cells and morphological atypia of the gastric epithelium. Furthermore, the HBV-exposed patients had lower programmed cell death protein 1 (PD-1) expression levels. Notably, information on Hp infection and antivirus treatment records was unavailable during the analysis in the abovementioned studies, which could have led to selection bias and obscured the actual connection between these two diseases. Therefore, there is a lack of robust evidence justifying the further bench-to-bed step for screening and preventing HBV-associated gastric cancer. Interestingly, recent evidence from a cross-sectional study in Iran (50) revealed that HBV infection was only associated with gastritis (OR = 12.46, 95%CI: 3.01–51.61) but not cancer (OR = 2.13, 95%CI: 0.24–18.70). Hp infection was more frequently detected in HBsAg-seropositive patients than in HBsAg-seronegative patients (p = 0.029). While the authors compared the clinicopathological characteristics of HBV infection patients with or without concurrent Hp infection, the study limitations, which included the study design and small sample size, could not be overlooked when concluding on the association between HBV infection and gastric cancer.

Most recently, two meta-analyses based on epidemiological studies reported consistent evidence on this topic. Wang et al. (51) determined that patients with gastric cancer presented a significantly higher HBV infection prevalence (OR = 1.39, 95%CI: 1.11–1.75) after patients with known Hp or EBV infections had been excluded. Yang et al. (53) reported that HBV infection was associated with a remarkably higher gastric cancer risk as compared with the healthy controls (HR = 1.26; 95%CI: 1.08–1.47).

Generally, the evidence supporting the positive relation between HBV infection and gastric cancer risk was mainly based on retrospective case–control and cohort studies. The confounding variables, which included other virus infections and cirrhosis, was not well controlled in many studies. Additionally, the underlying mechanisms of HBV-induced carcinogenesis in gastric cancer remain unknown.

### 3.2 Pancreatic carcinoma

While pancreatic carcinoma incidence is much lower than that of other gastrointestinal tumors, a worse survival outcome is predicted for patients with pancreatic carcinoma (69). Virus and autoimmune disease-induced inflammation is pivotal in pancreatic carcinoma development (70–72). Nevertheless, the role of HBV infection in pancreatic carcinoma risk remains uncertain. Initially, Hassan et al. (70) confirmed the apparent association between past exposure to HBV infection and pancreatic carcinoma risk in a US population (anti-HBc-seropositive patients: _adjusted_OR = 2.5; anti-HBc and anti-HBs double-seropositive patients: _adjusted_OR = 2.3; anti-HBc-seropositive but anti-HBs-seronegative patients: _adjusted_OR = 4). However, de Gonzalez et al. noted that the study lacked HBsAg testing results, which are considered a sensitive marker of chronic HBV infection. This absence might have led to uncertainties in directly evaluating the potential association between HBV infection and pancreatic cancer risk (73). Notably, de Gonzalez et al. presented evidence derived from a Korean population-based study (74). Interestingly, the findings did not support the strong association suggested by Hassan et al. (70), where HBsAg was considered the basis for diagnosing HBV infection (74). Furthermore, the results reported in the following works are non-concordant. In 2013, a notable meta-analysis that involved nine studies (60) reported more robust clinical evidence supporting the positive association between HBV infection and pancreatic cancer risk (_pool_OR = 1.28, 95%CI: 1.18–1.48). Other hospital-based studies reported similar results (36, 75). Most recently, a large-scale prospective cohort study also reported a direct association between HBV infection and pancreatic cancer onset (35). By contrast, two retrospective population-based cohort studies and one hospital-based study did not observe this positive correlation (54–56). Notably, each study could have been influenced by confounding factors that included the different pancreatic cancer histological classifications (pancreatic adenocarcinoma, neuroendocrine, or unspecified pancreatic tumors), different ethnic populations, and HBV infection history duration.

A previous study reported that patients with pancreatic cancer had remarkably increased HBV DNA and anti-HBc levels when compared with healthy controls. The results indicated that HBV infection in the pancreatic tissues might be the underlying etiology of pancreatic carcinoma development (76). Similarly, HBV antigens and replicative sequences have been identified in extrahepatic organs, including the pancreas (6). For this reason, some scholars speculated that the existence of HBV in pancreatic tissue could cause chronic pancreatic inflammation such as that in hepatic tissues, with the possibility of progression to malignant transformation (77). Contrastingly, HBV infection patients with pancreatic carcinoma demonstrated different metastatic patterns and survival (44), where HBV infection increased the synchronous liver metastasis rate in the patients. Interestingly, chronic HBV infection patients nevertheless demonstrated better survival when compared with inactive HBsAg carriers (p = 0.039). The authors proposed possible explanations to aid understanding of this phenomenon, where the beneficial role of anti-HBV therapy and subsequent immunological and tumor microenvironment changes could specifically be contributed to these divergence progression patterns. The early detection and diagnosis of pancreatic cancer are challenging (78, 79). Emerging evidence demonstrated a higher rate of early detection of pancreatic cancer in HBV infection patients under HCC surveillance (80). Therefore, whether routine screening for pancreatic conditions can be conducted during the surveillance of HBV-induced HCC is worth investigating.

### 3.3 Colorectal cancer

To date, compelling evidence supports the premise that HBV, especially chronic HBV infection status, can increase the colorectal cancer risk in populations from different regions. In a report from a large-scale Taiwan cohort, Kamiza et al. (11) determined that HBV infection patients exhibited a higher colon cancer development risk (_adjusted_HR: 1.36, 95%CI: 1.09–1.70). Several recent population-based epidemiological studies from different regions confirmed this potential correlation. Notably, in the China Kadoorie Biobank prospective cohort study from mainland China (13), HBsAg-seropositive patients had increased colorectal cancer risk (_adjusted_HR = 1.42, 95%CI: 1.12–1.81). However, this correlation was not validated in another two cohorts due to the limited sample size and cancer types. In line with the positive findings in gastric cancer, the Kailuan Cohort Study (61) also confirmed a positive association between HBV infection and colorectal cancer risk (whole group: _adjusted_HR = 1.77, 95%CI: 1.11–2.84; after excluding participants in whom HCC occurred within 1 year: _adjusted_HR = 2.05, 95%CI: 1.28–3.30). Epidemiologic investigations from Western countries presented more robust evidence of this correlation. Among chronic HBV infection patients in the Chronic Hepatitis Cohort Study (CHeCS), the patients with colorectal cancer had a slightly increased standardized rate ratio (SRR) of 1.50 (95%CI: 1.49–1.51) as compared with general health controls (National Program of Cancer Registries and Surveillance, Epidemiology, and End Results program cancer registries, 2006–2018) (14). A Canadian population-based study demonstrated that HBV infection patients had significantly elevated colorectal cancer risk (_adjusted_HR = 2.47, 95%CI: 1.85–3.28) as compared with uninfected patients (9). In patients with colorectal cancer, chronic HBV infection was observed as a pivotal risk factor of synchronous colorectal cancer liver metastasis and subsequent poor prognosis. By contrast, occult HBV infection instead of HBV DNA serum level decreased metachronous colorectal cancer liver metastasis occurrence with better overall survival (OS) and liver disease-free survival during the 5-year postoperative follow-up (45). Generally, these studies presented robust clinical evidence that chronic HBV infection can not only increase colorectal cancer risk but can also impair short-term survival in different ethnicities and regions. However, the underlying molecular mechanisms in HBV-induced colorectal cancer remain unknown. Furthermore, recent evidence demonstrated that anti-virus treatments in chronic HBV infection patients also increased the colorectal cancer risk (81). Accordingly, future analyses should consider this confounding information.

### 3.4 Head and neck cancer

HBV plays a role in head and neck cancer development and progression (82–85). Head and neck cancer with HPV-positive status is a specific head and neck cancer that has different tumor-node-metastasis (TNM) stage classifications and warrants varied treatment modalities (86). Recent epidemiological studies investigated the association between HBV infection and head and neck squamous cancer. Notably, Donà et al. (63) identified a positive association between HBV infection and head and neck squamous cancer in their case–control study. Alternatively, head and neck squamous cancer was more likely to coexist with chronic HBV infection (_adjusted_OR = 2.76; 95%CI: 1.64–4.64). Similar positive results were reported for Asian populations (43, 64). Nevertheless, the retrospective design of these studies meant that the exact causality between these two diseases could not be determined. In contrast, earlier evidence from a large population-based study did not support this correlation (87). Instead of HBV infection, patients with HCV infection presented a 2.28-fold higher oral cavity cancer risk as compared with the non-viral hepatitis group (6.15 *vs*. 2.69 per 10,000 person-years). Although some studies have examined the association between HBV infection and head and neck cancer, the evidence of the link between HBV and head and neck cancer occurrence is scanty and the mechanism of the high concurrent rate of HBV infection remains unclear.

### 3.5 Lymphoma

Emerging evidence confirmed that HBV demonstrates a significant capacity to infect peripheral blood mononuclear cells (PBMCs) and replicate in them (88, 89). For this reason, recent clinical studies focused on the association between chronic HBV infection and non-Hodgkin lymphoma, but reported inconsistent results (37, 38, 90–92). Notably, a comprehensive meta-analysis involving 58 studies and a total of 53,714 non-Hodgkin lymphoma cases and 1,778,591 non-cancer controls highlighted a remarkable association between HBV infection and increased B-cell non-Hodgkin lymphoma risk, specifically diffuse large B-cell lymphoma and follicular lymphoma (38). A recent hospital-based study also reported this correlation (37). Notably, the authors detected multiple integrated HBV DNA copies in lymphoma tissue *via* high-throughput viral integration detection. Correspondingly, the exons of four genes [fat atypical cadherin 2 (*FAT2*), senataxin (*SETX*), α10 integrin (*ITGA10*), lysosome-associated membrane protein-3 (*CD63*)] were interrupted by the HBV DNA, and the expression levels of seven HBV preferential target genes [ankyrin repeat and sterile alpha motif domain-containing protein 1B (*ANKS1B*), recombinant histone deacetylase 4 (*HDAC4*), fibronectin type III and laminin G-like domain-containing protein (*EGFLAM*), alpha1,2-mannosidase subtype IC (*MAN1C1*), XK-related 6 genes (*XKR6*), zinc finger and BTB domain-containing protein 38 (*ZBTB38*), coiled-coil domain containing 91 (*CCDC91*)] were significantly altered. In line with other HBV-associated cancers, HBsAg-seropositive diffuse large B-cell lymphoma exhibited unique clinicopathological characteristics that included younger age at diagnosis, more frequent spleen involvement, more advanced disease, and lower myelocytomatosis oncogene (*MYC*) expression rate when compared with the controls (91). However, HBV infection did not influence the treatment response of lymphoma. Consequently, more attention should be focused on the early screening of peripheral blood component disorders in chronic HBV infection patients. Furthermore, whether HBV infection can impair the survival of patients with non-Hodgkin lymphoma requires further exploration.

### 3.6 Esophageal cancer

Compelling evidence highlighted the fact that virus infection, particularly human papillomavirus (HPV) infection, could be involved in esophageal cancer development and progress (10, 93–97). Similarly, the role of HBV infection in esophageal cancer has been evaluated. The most recent meta-analysis, which involved 10 studies (142,437 cases and 1,382,254 controls) (10) confirmed that HBV infection patients had significantly increased esophageal cancer risk (_adjusted_OR = 1.19, 95%CI: 1.01–1.36). However, another hospital-based study (98) determined that HBV infection (referred to as HBsAg-seropositive) was a favorable prognostic factor for OS and disease-free survival (DFS) in patients with operable esophageal squamous cell carcinoma (DFS: _adjusted_HR = 0.79, 95%CI: 0.66–0.94; OS: _adjusted_HR = 0.80, 95%CI: 0.65–0.95). Some researchers speculated that HBV-induced immune microenvironment changes could contribute to this cancer progress divergence (99, 100). For example, the possession of specific genetic variants on the interleukin-17 (IL-17) gene might represent the underlying basis of the positive prognostic value of HBV infection on the survival outcomes of patients with esophageal cancer (101). These findings suggested the important but complex role of HBV infection in esophageal cancer. Unlike HPV-induced esophageal cancer, there remains a lack of robust evidence to explain the association between HBV infection and esophageal cancer. The histological examination of HBV-related indicators from esophageal epithelial cells might aid the exploration of detailed associations such as that in other gastrointestinal sites (52, 59, 77).

### 3.7 Breast cancer

Several studies have investigated the association between nutrition, infection, and environmental exposure with breast cancer risk (102–109); nevertheless, the correlations between HBV infection and breast cancer remain uncertain. Notably, a case–control study in China demonstrated that HBV infection was potentially an independent risk factor regardless of other factors in middle-aged breast cancer development (103), as the positive rate of HBc antibody (HBcAb) in patients with breast cancer (66.4%) was significantly higher than that in the controls (53.7%). Nevertheless, the serum HBsAg levels among the two groups were not markedly different. Another center reported significant associations between breast cancer and HBV infection, which could increase the breast cancer risk by approximately 16% (8). This correlation was validated in a US population-based epidemiological study (14). By contrast, two nationwide cohorts from China did not support the potential risk of HBV infection in inducing breast cancer (13, 107). Su et al. (107) determined that HCV infection but not HBV infection was more pivotal in breast cancer development. On the other hand, HBV infection patients with breast cancer had a higher risk for additional liver metastasis (102). Furthermore, recent studies indicated that chronic HBV infection patients with breast cancer tended to have an earlier tumor stage and higher histological grade (105), but survival outcomes were worse in young adult patients (104). These results supported precision treatment modalities for this subpopulation, and routine screening for HBV infection status could be a promising cost-effective means of preventing HBV reactivation during treatment (106). Further studies are needed to clarify the underlying mechanisms of the association between HBV infection and this highly common endocrine-related cancer.

### 3.8 Lung cancer

Chronic viral infections are frequent comorbidities in patients with lung cancer. However, whether there are potential connections between virus infection and lung cancer development remains unclear, as there have been few studies on this topic. Based on previous findings, HBV infection has a limited role in lung cancer risk. For example, an epidemiologic study on pan-cancer incidence analysis in HBV patients revealed only a slightly increased SRR on lung cancer risk in HBV conditions when compared with the general population (adjusted incidence: 105.8 *vs*. 90.4, SRR = 1.17, 95%CI: 1.16–1.18) (14). Similarly, no association was identified with elevated lung cancer risk in an HBsAg-seropositive Chinese population (35).

Recent studies focused on evaluating the prognostic outcomes of chronic HBV infection patients with NSCLC. Chronic HBV infection induces chronic inflammation. Chen et al. revealed that HBV infection patients with NSCLC presented unique survival patterns, which required more individualized clinical management (40, 110, 111). Combining HBV infection status with peripheral blood indicators, clinicians could obtain more precise survival predictions in this subpopulation. Serum HBV DNA levels could also be an independent prognostic factor in patients with NSCLC (41). HBV infection was recently associated with NSCLC immune expression (112, 113). HBV induced a systemic immune response with increased PD1 ligand 1 (PD-L1) expression levels in patients with NSCLC. However, a hospital-based study revealed that HBV infection might not affect the efficacy of immunotherapy (PD-1/PD-L1 inhibitors) in patients with advanced NSCLC. Nevertheless, the potential risk of virus reactivation when receiving treatment remains important, while the mechanism of HBV reactivation induced by immunotherapy therapy remains unclear (114, 115). For this reason, active surveillance before and during immunotherapy for HBV-associated serum indicators is important regardless of the rare incidence of these events.

### 3.9 Cervical cancer

Generally, HBV and HPV are DNA viruses that almost invariably integrate into the host genome in cancers (116, 117). The infections by both viruses present relatively the same clinical progress and the activated immune response can eradicate the viruses in most infected patients. However, a small sub-group can progress to carcinoma years later. The association between HPV infection and cervical cancer development has been established, where the evidence demonstrated a high concurrent rate of HBV and HPV co-infection (118, 119). Subsequently, recent hospital-based and epidemiological studies investigated the influence of HBV infection on cervical cancer development and prognosis (8, 12, 120–122). However, the influence of HBV infection on cervical cancer risk is debated. A large-scale hospital-based study from Korea (sample = 2,370 cervical cancer cases with an HBsAg-seropositive rate of 4.98%) (8) revealed a similar positive connection between HBV infection and cervical cancer risk as in other extrahepatic cancers (_adjusted_OR = 1.49). Conversely, this correlation was not reported in another larger-scale study from China (sample = 4,106 cervical cancer cases with an HBsAg-seropositive rate of 12.1%) (12). Moreover, a relatively lower risk for cervical cancer development was observed in the elderly (age > 60 years) HBV infection subpopulation (_adjusted_OR = 0.73). Nevertheless, the antivirus treatment rates among HBV infection groups in these two studies were unknown. Therefore, the conclusion for the causal effect between HBV infection and cervical cancer risk requires further evaluation with more comprehensive clinical information. An increasing number of studies revealed that anti-HBs seropositivity and prior HBV infection might be independent favorable prognostic factors for the OS and DFS of patients with cervical cancer (120, 121), whereas HBx protein exerted an adverse effect on cervical cancer progression (122). Accordingly, in-depth explorations of the interaction between HBV and HPV in inducing the carcinogenesis of cervical lesions and the immune microenvironment in HPV infection patients with cervical cancer with prior HBV infection could aid understanding of the role of HBV infection status and anti-HBV treatment in cervical cancer risk and future prognosis.

### 3.10 Nasopharyngeal carcinoma

A region-specific cancer, nasopharyngeal carcinoma (NPC) is frequently observed in Southern China, which accounts for approximately 50% of patients worldwide (age-standardized incidence rate of NPC in China in 2018: 3.0 per 100,000) (123, 124). NPC development likely involves close interplay between multiple factors, including EBV infection, environmental exposure, and genetics (123). Recent clinical studies assessed whether HBV infection played a role in NPC development. For example, a case–control study revealed a higher prevalence of anti-HBcAg seropositivity in patients with NPC as compared with patients with other benign tumors (47.26% *vs*. 39.33%) and anti-HBcAg seropositivity was an independent indicator of higher NPC risk (_adjusted_OR = 1.40). Moreover, patients with anti-HBcAg and EBV double-positivity had the highest NPC development risk (_adjusted_OR = 141.82) (62). While the role of HBsAg was not associated with NPC risk, another Southern China study revealed the pivotal role of HBsAg seropositivity in the prognosis of patients with NPC. That is, HBsAg-seropositive patients with NPC had worse outcomes, and a higher incidence of distant metastasis, specifically liver metastasis, was more common in such patients (125). Nevertheless, the evidence supporting the prediction value of HBV-associated serum indicators in NPC development was based on limited clinical hospital-based studies, and the underlying mechanisms remain unclear. Recent research evaluated the value of HBV infection on NPC prognosis (126, 127). Although HBsAg seropositivity was a negative prognostic factor in NPC, only patients with a particular tumor stage were significantly affected by this condition. In a Southern China report, Liu et al. (126) demonstrated that HBsAg seropositivity was an independent adverse prognostic predictor for survival and recurrence in patients with locoregionally advanced NPC but not patients with early-stage NPC. Interestingly, another recent study (127) from a similar Southern Chinese region indicated that HBsAg seropositivity markedly impaired the OS, DFS, and metastasis-free survival of patients with early-stage NPC. Furthermore, immune dysfunction conditions were observed in the HBsAg-seropositive subpopulation, which had a lower CD4^+^ T cell count than the HBsAg-seronegative group. The authors also identified a statistically significant beneficial role of anti-HBV treatment in recurrence-free survival (RFS) and metastasis-free survival of the patients.

During the past few years, immunotherapy (particularly PD-1/PD-L1 inhibitors) has become an important treatment strategy against solid tumors (128–130). Immunotherapy was also pivotal in patients with recurrent and/or metastatic NPC (131–133). While recent studies evaluated immunotherapy safety and efficacy among different cancers with HBV infection, research on this topic in patients with NPC is scarce (134–136). A meta-analysis that involved six studies revealed no significant difference in PD-1/PD-L1 inhibitor efficacy on the objective response rate among patients with HBV^+^HCC and HBV^-^HCC. However, the HBV^+^HCC group had a lower disease control rate (136). The association between adjuvant treatment in cancer and HBV reactivation was recently investigated at Chinese medical centers (137, 138). As expected, the results supported the premise that regular monitoring of the serum HBV indicators in HBV infection patients with NPC during treatment courses is warranted. Nevertheless, future studies are needed to quantitatively analyze the safety, efficacy, and cost-effectiveness of these treatment modalities.

## 4 Molecular pathway in carcinogenesis

HBV is a DNA virus with partly double-stranded relaxed circular DNA (33, 139). HBV infection is a pivotal pathogenesis in HCC development and the subsequent disease progression, and includes epigenetics and metabolic regulation, immune microenvironment dysfunction, HBV DNA integration, gene mutation, and cancer-promoting signaling pathway activation (3, 33, 116, 136, 140–142). Nevertheless, studies on HBV-associated extrahepatic cancers are relatively scarce. Previous study findings supported the premise that HBV might share similar mechanisms of HCC induction in inducing extrahepatic tissue carcinogenesis (103, 116, 122, 140, 141, 143).

### 4.1 HBV DNA integration

Genomic instability is a pivotal and frequent hallmark of cancer that stems from DNA repair gene mutations and drives cancer development (144, 145). The dynamic nature and unique features of chronic HBV infection such as viral variants, HBV DNA integration into host chromosomes, and extrahepatic reservoirs can induce genome instability to cause cellular transformation and subsequently tumorigenesis (3, 146, 147). Enhanced HBV molecular data and replication were recently identified in genes associated with oncogenic consequences in PBMCs (148). Although previous epidemiological studies determined that chronic HBV infection was significantly associated with extrahepatic cancer development, the exact causalities among the two diseases were not well documented. Given the remarkable advances in whole-genome, transcriptome, and exome sequencing technologies and genome-wide association studies (GWAS), some researchers preliminarily evaluated the role of HBV infection and cancer risk at the genetic level. Khoury et al. (141) reported that RNA sequencing results from The Cancer Genome Atlas (TCGA) database revealed two pronounced gene mutations [telomerase reverse transcriptase (*TERT*) and mixed-lineage leukemia 4 (*MLL4*)] in mediating HBV-associated HCC. Zapatka et al. (116) determined that viral integration was associated with local variations in genomic copy numbers and RNA sequencing demonstrated significant exclusivity between HBV infection and mutations in catenin beta-1 (*CTNNB1*), tumor protein p53 (*TP53*), and AT-rich interaction domain 1A (*ARID1A*). Furthermore, next-generation sequencing analysis identified five frequently mutated protein-coding genes in HBV-infected gastric cancer samples [histone-lysine N-methyltransferase 2B (*KMT2B*), histone-lysine N-methyltransferase 2D (*KMT2D*), sex-determining region Y box protein 1 (*SOX1*), fibroblast growth factor 12 (*FGF12*), and tubulin beta-2B chain (*TUBB2B*)] (90).

Mendelian randomization (MR) is an analytical approach that can improve causal inference by using genetic single-nucleotide polymorphisms (SNPs) as instrumental variables to infer the causality of exposure to an outcome. In MR analysis that used millions of SNPs in HBV and extrahepatic cancers, Kamiza et al. (143) confirmed that chronic HBV infection had causal effects on cervical cancer and gastric cancer in populations of Asian descent. By contrast, no association was observed between HBV infection and other extrahepatic cancers such as pancreatic and colorectal cancer in these populations. Notably, the SNP data are updated annually and potential connections might be identified in future analysis (149, 150). A recent genome-wide virus-integration analysis (151) revealed that three most common viruses (HBV, HPV, EBV) shared the same insertional mutagenesis (mainly mediated *via* a synthesis-dependent end-joining DNA repair mechanism). This finding could aid more in-depth understanding of the role of HBV infection in the development of other virus-associated cancers. So far, limited genetic data support the direct association between chronic HBV infection and extrahepatic cancer risk. Nevertheless, this field is worth exploring, which could enhance clinical observational findings and subsequent cancer prevention.

### 4.2 Chronic inflammation and the immune microenvironment

**Figure 3:**
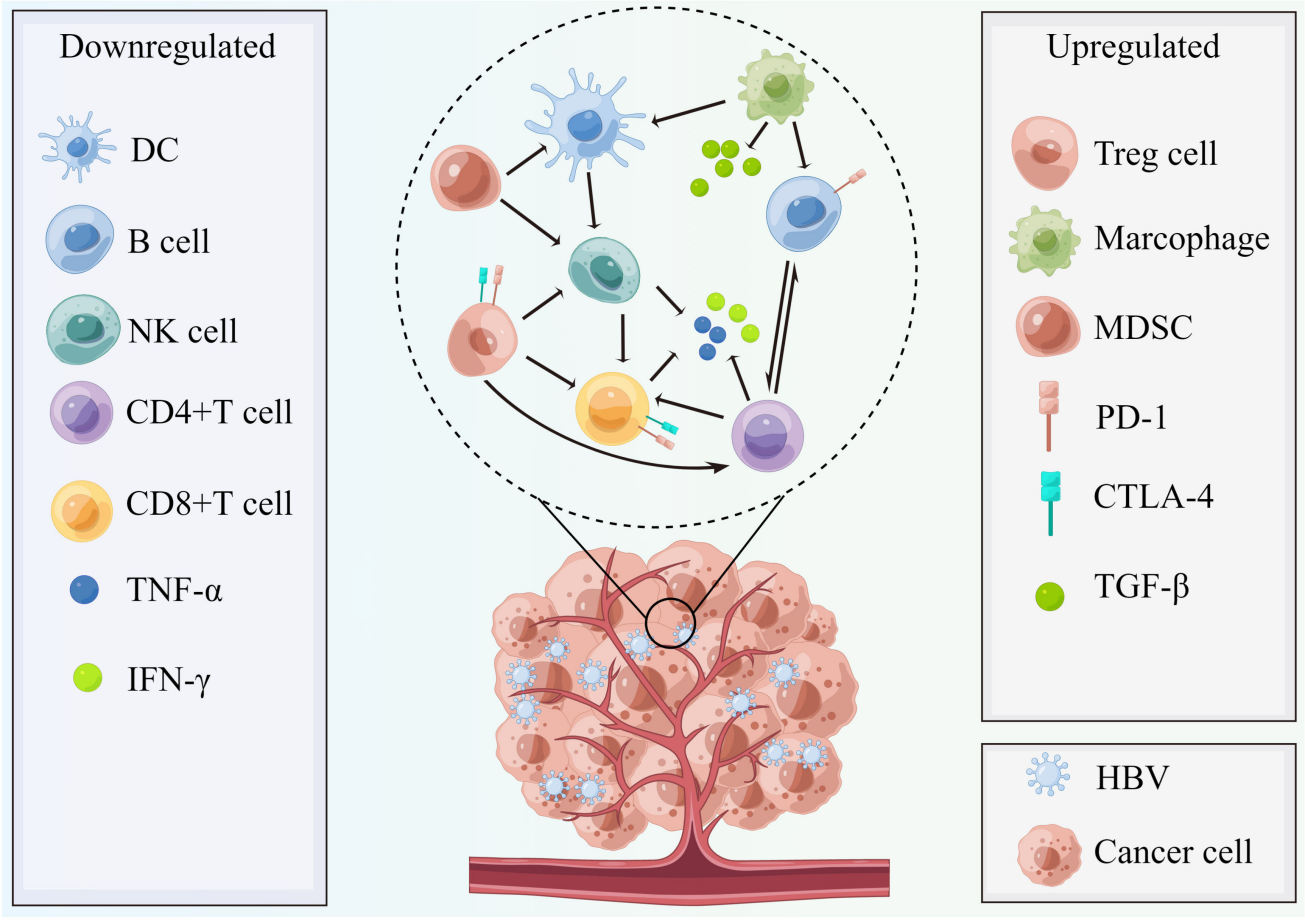
The chronic HBV-mediated tumor microenvironment with characteristics of more immunosuppressive conditions. In HBV infection conditions, the host immune status becomes more immunosuppressive and is characterized by weakening of stimulatory signal (DC, TNF-α, and IFN-γ), enhancement of inhibitory signals (TGF-β), decreased quantity of NK cells, and B cells, as well as effector T cells (CD4+ and CD8+T cell) and enrichment of Tregs cells, myeloid-derived suppressor cells, and M2 macrophage polarization. Therefore, the activated PD-1/PD-L1 pathway plays a suppressive role to help cancer cells’ immune escape and invasion and the CTLA-4 pathways also contribute to this process. This figure was drawn with the tool “Figdraw” by Dr. Yu Min. DC, dendritic cells; MDSC, myeloid-derived suppressor cells; PD-1, programmed cell death protein 1; CTLA-4, cytotoxic T-lymphocyte–associated antigen; TGF-β, transforming growth factor-β; NK cells, nature killer cells; TNF-α, tumor necrosis factor-α; IFN-γ, interferon γ; HBV, hepatitis B virus.

The intrahepatic inflammation induced by long-term chronic HBV infection significantly alters the host immune system, which is characterized by more immunosuppressive conditions given the weakening of co-activation signals, enhancement of co-inhibitory signals, functional impairment, decreased numbers of effector T cells such as HBV-specific CD8^+^ T cells but enrichment of T regulatory (Treg) cells (17, 152–160) (**Figure 3**). The most recent evidence confirmed that the adaptive immune response plays a major role in inducing both viral clearance and liver disease, whereas little innate immune activation was observed in the HBV condition (154). Therefore, the morbidities in chronic HBV infection patients include liver fibrosis and cirrhosis, where even cancers frequently develop in the subsequent years (4), partially owing to the intrahepatic immune disorders of T cell-induced immunopathology. However, the prior changes in the intrahepatic microenvironment of HBV infection contribute to the hepatic carcinogenic process and progress (160). A multidimensional immune landscape analyses revealed a distinct immune microenvironment in HBV-associated HCC that featured more immunosuppressive and exhausted phenotypes when compared with non-HBV-induced HCC (157). Furthermore, the immunopathological results in HBV mouse models revealed that hepatocellular priming of naïve HBV-specific CD8^+^ T cells led to their local activation and proliferation, but these cells did not further differentiate into effector cells (161, 162).

The most recent reviews demonstrated a noteworthy modification of the remaining immune components in the HBV-induced HCC tumor microenvironment (136, 160), with enhanced myeloid-derived suppressor cells (MDSC) cells and macrophages (M2 polarization) but inhibited dendritic cells and natural killer (NK) cells. In HBV-associated HCC, the elevated Treg cell levels aided the maintenance of a tolerogenic environment in the liver and suppressed the proliferation and interferon γ (IFN-γ) production of autologous PBMCs mediated by the HBV condition (163), which were significant immune alterations. Notably, single-cell sequencing results demonstrated that tumor-associated macrophages (TAM) significantly inhibited tumor T-cell infiltration, and T cell immunoreceptor with Ig and ITIM domains–nectin cell adhesion molecule 2 (TIGIT–NECTIN2) interaction regulated the immunosuppressive environment (153). B cells are a major tumor microenvironment component that are predominantly associated with tertiary lymphoid structures. Sufficient evidence demonstrated the double-edged sword of B cells with different phenotypes in cancer occurrence and progress (164). Initially, B cells defend against chronic HBV infection by producing humoral antibodies (136, 165). In chronic HBV infection, B regulatory (Breg) cells and secreted cytokines such as IL-10, IL-35, and transforming growth factor-β (TGF-β) aid the construction of the immune-suppressive landscape in the liver. Therefore, the immune escape caused by the intrahepatic immunosuppressive condition subsequently increases the risk of HBV-induced HCC (155–157). To date, research focused on the tumor-associated immune microenvironment of HBV-induced extrahepatic cancers remains scarce. Nevertheless, immunotherapy safety and efficacy in patients with cancer concurrent with HBV infection were evaluated recently (112, 113). Notably, patients with NSCLC concurrent with HBV infection had a significantly higher percentage of PD-L1 expression when compared with patients without HBV infection. The safety analysis revealed that HBV infection status did not impair the immunotherapy efficacy in patients with NSCLC. Rather, the HBV groups demonstrated more durable clinical benefits and longer progression-free survival and OS than the non-HBV group (113). For this reason, these findings indicate viral mediation of a systemic immune response, which could potentially affect PD-L1 expression-guided immunotherapy in HBV infection patients with extrahepatic cancer. Nevertheless, whether patients with HBV-associated extrahepatic cancers can derive more benefit from immunotherapy requires further exploration. The evaluation of peripheral blood immune cell subsets and single-cell sequencing-related immune infiltration scores aid improved individualized treatment modalities for both HBV-mediated HCC and non-HCC cancer subtypes.

### 4.3 HBx protein

The detailed biological mechanism linking chronic HBV infection with extrahepatic cancers has not been fully illustrated. Some studies reported elevated HBx protein expression levels and virus DNA copies in several extrahepatic tissues, especially the gastrointestinal system, which suggested that HBV can initiate and promote tumorigenesis outside the liver (5, 6). Currently, it is thought that the transcriptional coactivator HBx protein is crucial in initiating tumorigenesis by modulating key apoptosis regulators, which interferes with the DNA repair pathways and tumor suppressor genes (122, 166–170). Furthermore, HBx protein significantly caused host chromosomal instability (171). Therefore, the target downstream molecules in HBx protein mediate cell carcinogenesis, proliferation, viability, and migration by modulating multiple signaling pathways (167, 170, 172, 173). Specifically, Lei et al. (172) demonstrated that ARRB1 (arrestin β1) was pivotal in HBV-related HCC by modulating autophagy and the CDKN1B–CDK2–CCNE1–E2F1 signaling pathway. However, evaluation studies on HBx protein and extrahepatic cancer occurrence are limited. Notably, Song et al. (13) observed higher HBx protein expression in the cancerous part of the tissue specimen than in the healthy part of the same specimen among several gastrointestinal cancers, which supported the oncogenic role of HBx protein. Moreover, a recent report stated that HBx protein promoted pancreatic cancer development *via* the PI3K–Akt signaling pathway (166). Based on these non-negligible connections between HBx protein and intrahepatic and extrahepatic cancers, these findings could guide the design and interpretation of subsequent clinical and experimental studies to identify more potential cancer prevention and treatment targets. Furthermore, the low HBx expression levels during HBV infection and the lack of satisfactory detection strategies mean that these findings require verification in models that more closely mimic HBV infection and HBV-associated cancers.

### 4.4 Cancer-related signaling pathway activation

**Figure 4:**
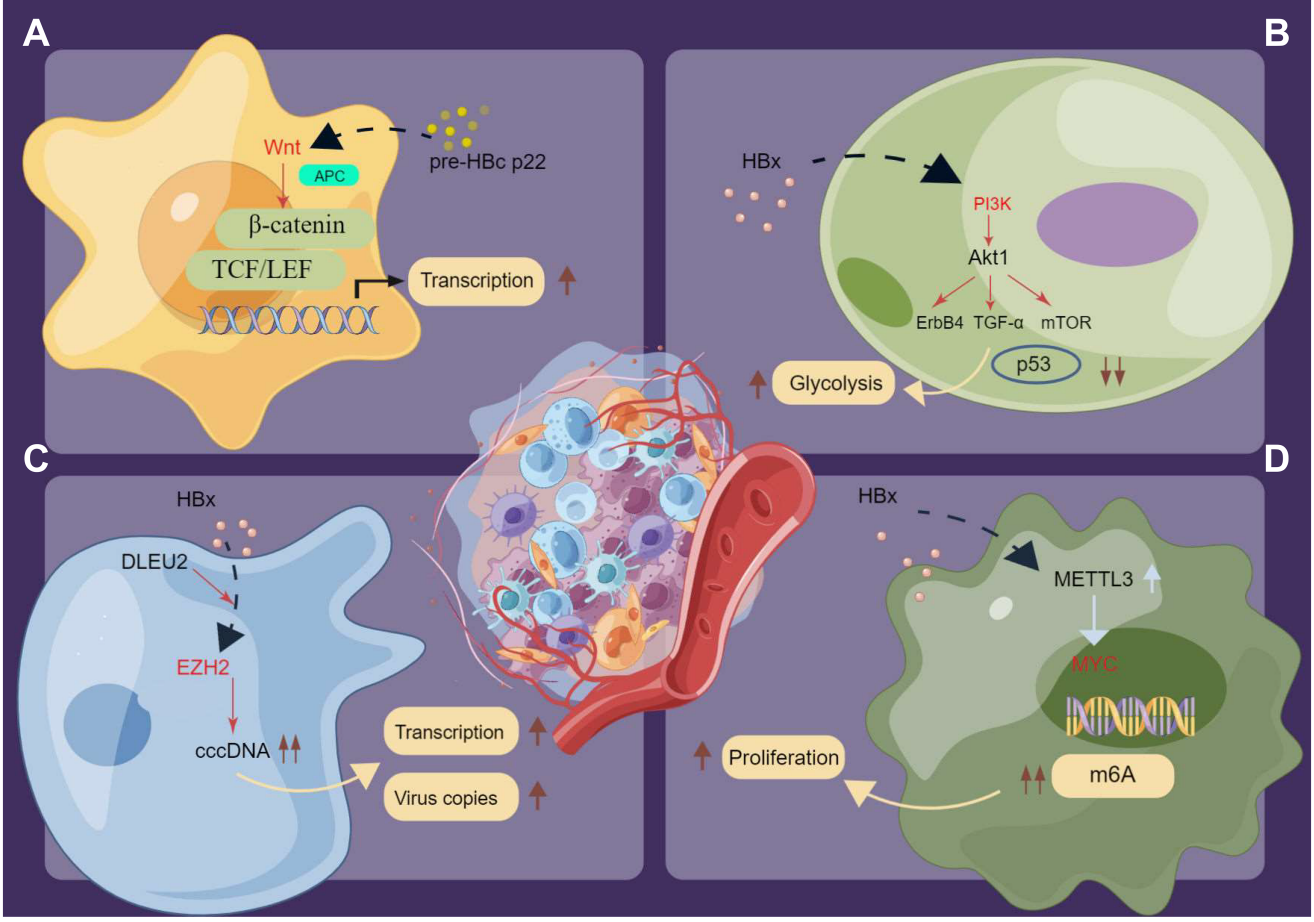
The potential signaling pathways in inducing extrahepatic cancers under the chronic HBV infection. **(A)** HBV p22 elevates TCF/β-catenin transcription in cancer cells owing to mutations in downstream genes of the Wnt pathway. **(B)** HBx activates the PI3K/Akt signaling pathway and promotes glucose metabolic reprogramming in extrahepatic cancers. **(C)** HBx enhances the transcription and induces the accumulation of DLEU2 in infected hepatocytes. The co-recruitment HBx protein and DLEU2 could elevate the expression levels of EZH2 as well as the levels of viral cccDNA and further boost transcription and viral replication in the host. **(D)** HBx increases the expression level of oncogenes like MYC and the N6-methyladenosine (m6A) modification of MYC mRNA to promote the proliferation of cancer cells. Pre-HBc p22, pre-hepatitis B core protein 22; HBx, Hepatitis B virus X protein; TCF/LEF, T-cell Factor/Lymphoid Enhancing Factor; TGF-α, Transforming Growth Factor α; cccDNA, covalently closed circular DNA; DLEU2, deleted in lymphocytic leukemia 2; EZH2, zeste homolog 2; MYC, myelocytomatosis oncogene; m6A, N6-methyladenosine.

Over the past few years, compelling evidence indicated that HBV infection induces numerous cancer-related signaling pathways in hepatocellular tissue carcinogenesis (167, 174–178). On the contrary, evaluation data on the underlying molecular mechanisms of chronic HBV infection-associated extrahepatic cancers remain scarce. Nevertheless, some potential pathways described in the literature could aid understanding of the complex mechanisms of HBV infection-mediated extrahepatic cancer occurrence (**Figure 4**). Notably, Tran et al. (179) used mouse models and determined that HBV pre-core protein p22 alone could activate Wnt signaling. Furthermore, HBV p22 elevated TCF/β-catenin transcription in colon cancer cell lines (SW480 and HCT116) owing to mutations in downstream genes of the Wnt pathway, namely adenomatous polyposis coli (*APC*) and *CTNNB1* (**Figure 4A**). Similarly, disordered Toll-like receptor 7 and 9 signaling was observed in chronic HBV infection (142). HBx protein activates various signaling pathways in HBV-associated extrahepatic tissue carcinogenesis (166, 168, 169). Specifically, the basic study by Chen et al. (166) demonstrated that HBx increased pancreatic cancer risk by modulating the PI3K–Akt signaling pathway, with elevated expression of human epidermal growth factor receptor 4 (ErbB4) and TGF-α in parallel with HBx protein expression (**Figure 4B**). PI3K–Akt pathway activation was also observed in gastric cancer samples *in vivo* and *in vitro* (168). Furthermore, p53 pathway impairment was observed in gastric cancer samples with HBx protein integration, which promoted glucose metabolic reprogramming in gastric tissue and subsequent cancer development (168). Salerno et al. (170) demonstrated that HBx protein enhanced gene transcription and induced the accumulation of the long non-coding RNA (lncRNA) deleted in lymphocytic leukemia 2 (DLEU2) in infected hepatocytes. The co-recruitment of HBx protein and DLEU2 elevated histone methyltransferase enhancer of zeste homolog 2 (EZH2) expression levels and viral covalently closed circular DNA (cccDNA) levels and enhanced transcription and viral replication in the host (**Figure 4C**). Moreover, a recent study (169) highlighted that HBx might increase the MYC expression level by inducing the methyltransferase-like protein 3 (METTL3)-mediated N6-methyladenosine (m6A) modification of *MYC* mRNA, which promoted gastric cancer onset and progression in preclinical analysis (**Figure 4D**). Generally, while several *in vivo* and *in vitro* studies identified the potential different signaling pathways in gastrointestinal cancer occurrence, whether these signaling pathways are also involved in other extrahepatic cancers was not fully investigated. Moreover, the effects of epigenetic modifications in the activation or blocking these signaling pathways of chronic HBV-induced extrahepatic cancers require further investigation.

## 5 Treatment and prognosis

Currently, there remains a lack of specific treatment guidelines for HBV-mediated extrahepatic cancers. Therefore, the established approach is to address the primary malignancy and monitor liver function and the HBV-associated indicators during treatment. Alternatively, clinicians are more interested in assessing the potential virus reactivation risk during novel adjuvant therapy, especially immunotherapy (113–115, 135, 137, 138). Immunotherapy might not significantly increase HBV reactivation risk, but regular monitoring of HBV DNA copies and antiviral prophylaxis could aid the prevention of this potential complication (113, 138). Most studies highlighted that antiviral therapy can reduce cancer metastasis risk and recurrence and subsequently improve long-term survival (42, 127, 180). Additionally, drug repurposing is a recent hot topic in the design of novel anti-cancer treatment modalities (181–183). Notably, Choi et al. (184) demonstrated in the HBV population that statins significantly reduced HBV-associated HCC risk with a dose-dependent effect. Preclinical studies reported that atorvastatin downregulated the HBx protein-induced Akt pathway *via* the purinergic receptors (P2X) and further decreased hepatocyte proliferation and invasiveness (185). Other studies suggested that anti-platelet therapy contributed to inhibiting HCC development by limiting the intrahepatic accumulation of HBV-specific CD8^+^ T cells (154, 186). These findings on decreasing the risk of HBV-mediated HCC can also aid exploration of the role of drug repurposing to prevent extrahepatic cancers.

There is no consensus regarding the role of HBV infection in the clinical prognosis of different cancers. Regarding the lymph nodes and distant organ metastasis, a cohort study from an endemic area in China demonstrated that HBV infection decreased the liver metastasis risk in patients with colorectal cancer but did not influence survival (187). Additionally, a pooled meta-analysis involving 11 studies suggested that HBV infection was associated with a lower rate of lymph node metastasis, better DFS, and prolonged OS in patients with intrahepatic cholangiocarcinoma and esophageal cancer (98, 188). By contrast, higher serum titer HBc antibody was recently identified as an adverse factor in increasing liver metastasis risk and poor survival probability in colorectal cancer (189). Similarly, HBsAg seropositivity was a biomarker that predicted worse survival of early-stage NPC (127). It should be noted that most of the existing evidence on this topic was derived from Asian populations (188). Whether these correlations between HBV biomarkers and varied cancer prognosis can be generalized to other ethnicities and regions requires validation. Furthermore, the distinct roles of HBc, HBsAg, and other serum biomarkers in cancer outcomes indicate that further studies are warranted to illustrate the landscapes of altered host immune function and the gene expression profiles under the altered hepatic microenvironment in HBV. Such findings would aid precision medical decision-making to manage these unique cancer populations.

## 6 Prospects

Currently, there is no guideline to support HBV screening in general patients with cancer. Nevertheless, it may be likely that possible target cancers (determined by their associations with HBV) can be regularly included in the routine surveillance programs of chronic HBV infection patients. Nonetheless, the cost-effectiveness of the proposed policies must be determined before actual clinical application. More importantly, the safety and feasibility of antiviral treatment in the early prevention and survival of HBV infection patients with extrahepatic cancer are also important tasks in future studies. Some key population groups, specifically people living with HIV, have remarkably higher HBV infection prevalence than the general population (27, 118, 190). Notably, much clinical epidemiological data demonstrated significantly increased extrahepatic cancer risk in hepatitis virus and HIV co-infection subpopulations (9, 191). Based on these findings, these key populations with HBV infection may derive more benefits from targeted cancer prevention and diligent clinical monitoring. Furthermore, future studies are warranted to validate the clinical findings and clarify the associated mechanisms, especially for the immunosuppressed population.

## 7 Conclusions

The clinical epidemiological evidence supports the premise that chronic HBV infection significantly increases the risk of several extrahepatic cancers, especially gastrointestinal cancers. Although compelling studies confirmed the underlying mechanisms in HBV-mediated HCC involving different molecular signaling pathways, there are few explorations of HBV-mediated extrahepatic cancers. Therefore, future studies are warranted to fill these gaps and provide more insightful evidence to aid understanding of the interactions among HBV infection-derived immune microenvironment changes, epigenetic modification, elevated cancer-promoted signaling pathways, serum biomarkers, and the oncogenesis of extrahepatic organs.

## Author contributions

XH and XP were responsible for the study’s concept and design. YM and XX did the data and project management. ZL and YM did the literature search and analysis. YM, ZL, RL, ZW and JJ interpreted the data. YM, XW, XX and XP drafted the manuscript. All authors contributed to the article and approved the submitted version.

## Glossary

**Table:**

HBV	hepatitis B virus
HCC	hepatocellular carcinoma
HbcAg	hepatitis B core antigen
HbeAg	hepatitis B e antigen
HbcAb	hepatitis B core antibody
R/M	recurrent/metastatic
TNM	Tumor-Node-Metastasis
FAT2	fat atypical cadherin 2
SETX	senataxin
ITGA10	α10 integrin
CD63	lysosome-associated membrane protein-3
ANKS1B	Ankyrin repeat and sterile alpha motif domain-containing protein 1B
HDAC4	recombinant histone deacetylase 4
EGFLAM	fibronectin type-III and laminin G-like domain-containing protein
MAN1C1	alpha1,2-mannosidase subtype IC
XKR6	XK related 6 gene
ZBTB38	zinc finger and BTB domain-containing protein 38
CCDC91	coiled-coil domain containing 91
DC	Dendritic cells
PBMCs	peripheral blood mononuclear cells
MDSC	myeloid-derived suppressor cells
PD-1	programmed cell death protein 1
CTLA-4	cytotoxic T-lymphocyte–associated antigen
TGF-β	transforming growth factor-β
NK cells	nature killer cells
TNF-α	tumor Necrosis Factor-α
ErbB4	human epidermal growth factor receptor-4
TERT	telomerase reverse transcriptase
MLL4	mixed-lineage leukemia 4
CTNNB1	catenin beta-1
DLEU2	deleted in lymphocytic leukemia 2
APC	adenomatous polyposis coli
TP53	tumor protein p53
ARID1A	AT-rich interaction domain 1A
KMT2B	histone-lysine N-methyltransferase 2B
KMT2D	histone-lysine N-methyltransferase 2D
SOX1	sex-determining region Y box protein 1
FGF12	fibroblast growth factor 12
TUBB2B	tubulin beta-2B chain
IFN-γ	interferon γ
Pre-HBc p22	pre-hepatitis B core protein 22
P2X	purinergic receptors
HBx	hepatitis B virus X
ARRB1	arrestin β1
TCF/LEF	T-cell Factor/Lymphoid Enhancing Factor
TGF-α	transforming Growth Factor α
cccDNA	covalently closed circular DNA
EZH2	zeste homolog 2
m6A	N6-methyladenosine
OR	odd ratio
HR	hazard ratio
SRR	Standardized rate ratio
HbsAg	hepatitis B surface antigen
CKB	China Kadoorie Biobank
HNC	head and neck cancer
NPC	nasopharyngeal carcinoma
HNSCC	head and neck squamous cell carcinoma
NHL	non-Hodgkin’s lymphoma.
